# Opposing Baltimore Medical Colleges—Correction

**Published:** 1886-02

**Authors:** 


OPPOSING BALTIMORE MEDICAL COLLEGES.—CORRECTION.—Under this title a communication appears in January No., 1886, of the *Dental Cosmos* written by the *Correspondent of Baltimore Times*, from which it is copied, to the effect that the State law of Pennsylvania demands that all medical students who graduate outside of the State shall have their diplomas indorsed by some recognized school in the State. It further states that "heretofore both Jefferson College and the University of Pennsylvania have indorsed such diplomas, Jefferson College only making exception in cases of irregular or bad schools." "But in order to more fully comply with the State law the University of Pennsylvania will hereafter demand that all students who want their diplomas indorsed must stand an examination before the faculty, and for such an examination pay $30."

As this statement might be understood as applying to the diploma of the University of Maryland, School of Medicine, it is only necessary to state that a letter recently received by Professor Wm. T. Howard, M. D., from Professor Roberts Bartholow, M. D., of Jefferson Medical College, Philadelphia, asserts that the diploma of the University of Maryland, School of Medicine, Baltimore, *will be indorsed* by Jefferson Medical College.

It will therefore be seen that the proposed action does not apply to the diploma of the University of Maryland, School of Medicine, which has always maintained a high and unquestionable reputation.

